# Study protocol: Cerebral autoregulation, brain perfusion, and neurocognitive outcomes after traumatic brain injury -CAPCOG-TBI

**DOI:** 10.3389/fneur.2024.1465226

**Published:** 2024-10-16

**Authors:** Juliana Caldas, Danilo Cardim, Philip Edmundson, Jill Morales, Aaron Feng, John Devin Ashley, Caroline Park, Alex Valadka, Michael Foreman, Munro Cullum, Kartavya Sharma, Yulun Liu, David Zhu, Rong Zhang, Kan Ding

**Affiliations:** ^1^University of Texas Southwestern Medical Center, Dallas, TX, United States; ^2^Bahiana School of Medicine and Public Health, Salvador, Bahia, Brazil; ^3^D'or Institute for Research and Teaching, Salvador, Bahia, Brazil; ^4^Texas Health Resources, Dallas, TX, United States; ^5^Baylor University Medical Center, Dallas, TX, United States; ^6^Albert Einstein College of Medicine, New York, NY, United States

**Keywords:** non-invasive neuromonitoring, dynamic cerebral autoregulation, near-infrared spectroscopy, moderate to severe traumatic brain injury, transcranial Doppler

## Abstract

**Background:**

Moderate–severe traumatic brain injury (msTBI) stands as a prominent etiology of adult disability, with increased risk for cognitive impairment and dementia. Although some recovery often occurs within the first year post-injury, predicting long-term cognitive outcomes remains challenging, partly due to the significant pathophysiological heterogeneity of TBI, including acute cerebrovascular injury. The primary aim of our recently funded study, cerebral autoregulation, brain perfusion, and neurocognitive outcomes after traumatic brain injury (CAPCOG-TBI), is to determine if acute cerebrovascular dysfunction after msTBI measured using multimodal non-invasive neuromonitoring is associated with cognitive outcome at 1-year post-injury.

**Methods:**

This longitudinal observational study will be conducted at two Level 1 trauma centers in Texas, USA, and will include adult patients with msTBI, and/or mild TBI with neuroimaging abnormalities. Multimodal cerebral vascular assessment using transcranial Doppler and cerebral near-infrared spectroscopy (NIRS) will be conducted within 7-days of onset of TBI. Longitudinal outcomes, including cognitive/functional assessments (Glasgow Outcome Scale and Patient-Reported Outcomes Measurement Information System), cerebral vascular assessment, and imaging will be performed at follow-ups 3-, 6-, and 12-months post-injury. We aim to recruit 100 subjects with msTBI along with 30 orthopedic trauma controls (OTC). This study is funded by National Institute of Neurological Disease and Stroke (NINDS) and is registered on Clinicaltrial.org (NCT06480838).

**Expected results:**

We anticipate that msTBI patients will exhibit impaired cerebrovascular function in the acute phase compared to the OTC group. The severity of cerebrovascular dysfunction during this stage is expected to inversely correlate with cognitive and functional outcomes at 1-year post-injury. Additionally, recovery from cerebrovascular dysfunction is expected to be linked to cognitive recovery.

**Conclusion:**

The results of this study could help to understand the contribution of cerebrovascular dysfunction to cognitive outcomes after TBI and pave the way for innovative vascular-focused interventions aimed at enhancing cognitive recovery and mitigating neurodegeneration following msTB. In addition, its focus toward personalized medicine to aid in the management and prognosis of TBI patients.

## Background and rationale

Nonfatal traumatic brain injury (TBI) stands as a prominent etiology of adult disability, presenting substantial morbidity for the victims, their families, and society at large, with several negative impacts, including cognitive impairment (1, 2). Moderate–severe TBI (msTBI), defined as a Glasgow Coma Scale (GCS) score of 3–12 on admission carries the largest burden of morbidity and mortality (2), with notable variability in clinical outcomes following msTBI. Cognitive impairment, including impaired memory, slow processing speed, and diminished attention span, is particularly prevalent after msTBI (3–5). Although some recovery typically occurs within the first year post-injury (3), accurately predicting long-term cognitive outcomes remains challenging.

This challenge is partly due to the high pathophysiological heterogeneity of TBI, particularly the occurrence of acute cerebrovascular injury that is common following msTBI (6). Acute cerebrovascular injury after msTBI includes vasospasm of larger cerebral arteries, trauma-induced vascular damage at smaller vessel levels, and disruption of the blood–brain barrier and endothelium due to shear (6, 7). In addition, the injury processes involve altered brain metabolism, impaired waste clearance, and neuroinflammation, leading to brain hypoperfusion, disrupted neurovascular coupling, impaired cerebral autoregulation (CA), as well as inefficient adjustments in cerebral blood flow to changes in perfusion pressure (8–10). There is some evidence suggesting that acute cerebrovascular dysfunction following TBI, as assessed by cerebral blood flow and CA by transcranial doppler (TCD), is associated with unfavorable functional outcomes in those with Glasgow Outcome Scale scores less than 3 (10). Nevertheless, a significant gap persists in the literature regarding longer-term follow-ups and comprehensive assessments of cerebrovascular function alongside cognitive function for this patient population.

The most significant recovery of cognitive function usually occurs during the first 6 months post-injury, but the patients may continue to experience improvement afterward (3, 11). The risk factors for poor cognitive recovery include older age, more severe initial injury, lower pre-injury work productivity, and lower discharge functional status (12). However, currently, none of these clinical factors can reliably predict cognitive outcomes following msTBI (12). Conversely, studies have suggested cerebrovascular dysfunction defined as persistent CA and regional brain perfusion alterations utilizing TCD is associated with lower cognitive performance in the chronic phase of TBI (13–17).

The literature for msTBI outcomes is limited by insufficient long-term follow-up durations and suboptimal evaluations of cognitive function. Additionally, the role of acute cerebrovascular injury and its recovery in relation to cognitive recovery has not been studied prospectively. The Cerebral autoregulation, brain perfusion, and neurocognitive outcomes after traumatic brain injury (CAPCOG-TBI) study is a prospective observational study funded by National Institute of Neurological Disease and Stroke (NINDS) and aims to investigate the association between acute cerebrovascular dysfunction after msTBI and cognitive outcomes at 1-year post-injury. Moreover, it seeks to assess longitudinal changes in cerebrovascular function focusing on dynamic cerebral autoregulation and brain perfusion, as well as its impact on cognitive recovery during the first year post-injury.

## Methods and analysis

### Aim of the study

Our primary research aim is to determine if acute cerebrovascular dysfunction after TBI is associated with cognitive outcome at 1-year post-injury. Secondly, to determine the temporal associations between the recovery of cerebrovascular function and cognitive outcomes after TBI. The third aim is to determine the temporal associations of acute cerebrovascular dysfunction and its recovery with imaging biomarkers of neurodegeneration after TBI.

**Figure 1:**
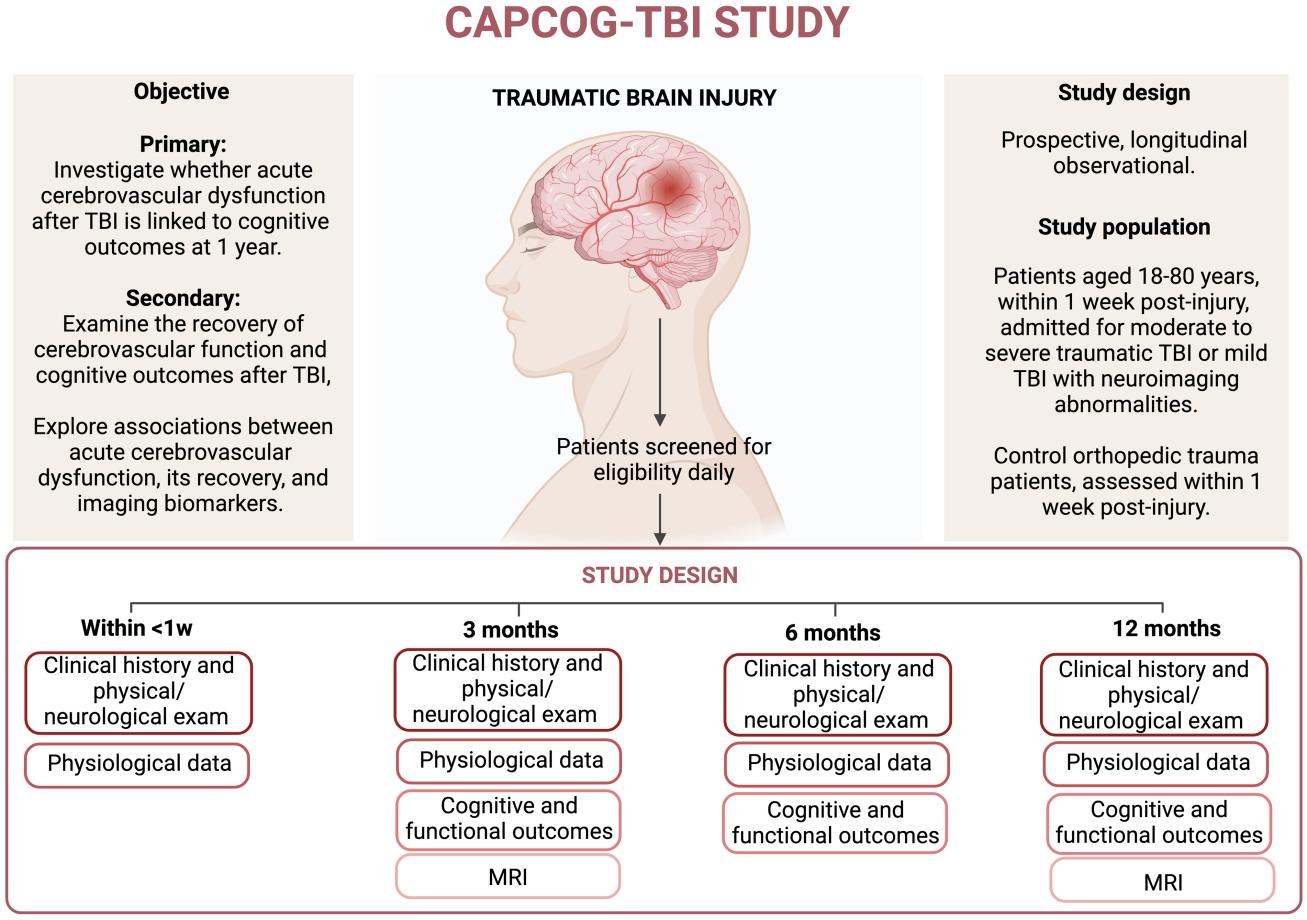
The figure illustrates the participant timeline. TBI, traumatic brain injury; MRI, Magnetic Resonance Imaging. * MRI will be performed at 3 and 12 months. **6 months follow-up will be only done in TBI group.

We expect that the severity of cerebrovascular dysfunction during the acute stage will be inversely associated with cognitive outcomes and functional outcomes at 1-year post-injury. Additionally, we also anticipate that, compared to an orthopedic control (OTC) group with no TBI history, the TBI patients will exhibit impaired dynamic CA in the acute phase. Assessments of CA during the acute stage of TBI are expected to independently predict functional and cognitive outcomes at 1-year post-injury. The results of this study could pave the way for innovative vascular-focused interventions aimed at enhancing cognitive recovery and mitigating neurodegeneration following TBI (Figure 1).

### Study design

This study will be a prospective, longitudinal observational study. We propose to recruit 100 subjects with TBI and 30 orthopedic trauma controls within the first week after the initial injury, matching them for age range and gender. We will follow these patients with physiological, imaging, and cognitive/functional outcome measures over a year (Figure 1).

#### Study population

Patients aged between 18 and 80 years old, within 1-week post-injury, and admitted to the hospital for moderate to severe TBI and/or mild TBI with neuroimaging abnormalities are eligible for inclusion (see Table 1). Additionally, individuals with orthopedic injury without TBI can participate as a control group if they meet the inclusion criteria outlined in Table 1 and do not meet any of the exclusion criteria.

**Table 1:** Eligibility criteria.

Inclusion Criteria for TBI Group A documented moderate to severe TBI posttraumatic Amnesia (PTA) > 24 h Loss of consciousness (LOC) >30 min Global cognitive score (GCS) <13, or Intracranial neuroimaging abnormalities (Head CT or MRI) Mild TBI with abnormalities on imaging exam. Ages 18–80 years-old 1 week post injury and admitted to the hospital for TBI. All participants must be fluent in English or Spanish. Inclusion Criteria for non-TBI Orthopedic Control Group: Abbreviated Injury Score of ≤4 (not life threatening) for extremity and/or pelvis injury and/or rib fracture Without evidence of TBI based on history or head CT 1 week post injury and admitted to the hospital for trauma. All participants must be fluent in English or Spanish. Exclusion Criteria for the study Significant polytrauma that would interfere with follow-up and outcome assessment. Major debilitating baseline mental health disorders, major debilitating neurological disease, impairing baseline awareness, cognition, or validity of follow-up and outcome assessment. Significant history of pre-existing conditions that would interfere with follow-up and outcome assessment (e.g., end-stage cancers). Patients on psychiatric hold. Prisoners or patients in custody, pregnancy, low likelihood of follow-up (e.g., participants or family indicating low interest, residence in another state or country, homelessness or lack of reliable contacts). Current participant in an interventional trial (e.g., drug, device, behavioral). Penetrating TBI, spinal cord injury with ASIA score of C or worse and contraindications to MRI.

### Recruitment plan

Participants meeting inclusion criteria will be recruited from Parkland Hospital and Texas Health Presbyterian Hospital Dallas. The research coordinator will identify potential subjects daily from various hospital units using electronic medical records and liaising with on-duty medical staff. After screening against criteria, eligible subjects will be approached for study participation.

### Informed consent

Prior to enrollment, the research team will evaluate participants’ competency for informed consent using the Galveston Orientation and Amnesia Test (GOAT). If the GOAT score is below 75, consent from a Legally Authorized Representative (LAR) will be sought and the research coordinator will obtain written permission from that person. All aspects of the study will be thoroughly discussed with patients and legal representatives, and an informed discussion will be undertaken. A copy of the informed consent will be provided to the patient and/or legal representative. For participants consented by an LAR in the acute phase, we will repeat the GOAT assessment and re-consent them during the 3-month follow-up visit.

#### Withdrawal from participation

The patients or their LAR may withdraw the subject from further participation at any time and for any reason. Consistent with Office for Human Research Protections (OHRP) and Food and Drug Administration (FDA) guidance, participant data collected prior to withdrawal from the study is maintained in the study database, but no additional participant data will be collected from the participant or their medical record after withdrawal from the study.

### Follow-up retention plan

The participant or the LAR will be approached at 3 months (±2 weeks), 6 months (±2 weeks), and 12 months (±1 month) for follow-up assessments (Figure 1). For follow-up, participants will be invited to come on 2 days’ visits. On the first day, the patient will undergo a physical neurological exam, as well as cognitive and functional assessments (Table 2). On the second day, a cerebral vascular assessment will be conducted. The MRI will be performed at the University of Texas Medical Center during the first day visit at 3 and 12 months (Figure 1). If participants are still hospitalized at these time points, the follow-up will take place there.

**Table 2:** Functional, cognitive and psychological health and quality of life.

Domain	Outcome measures	Time points	Interview type
Screening assessment			
	Speech intelligibility	3 M, 6 M*, 12 M	T, IP
	GOAT	3 M, 6 M*, 12 M	T, IP
Comprehensive assessment battery			
Cognition (primary outcome)	NIHTB cognitive battery	3 M, 6 M*, 12 M	IP
	Hopkins Verbal Learning Test (HVLT)	3 M, 6 M*, 12 M	IP
	Trail Making Test	3 M, 6 M*, 12 M	IP
	Processing Speed Index (WAIS-IV PSI)	3 M, 6 M*, 12 M	IP
	SWAPS (aka TAPS)	3 M, 6 M*, 12 M	IP
Global outcomes (secondary outcome measures)	Functional Status Examination (FSE)	3 M, 6 M*, 12 M	T, IP
	Revised Glasgow Outcome Scale Extended (R-GOSE)	3 M, 6 M*, 12 M	T, IP
	Expanded Disability Rating Scale Post-Acute Interview (E-DRS-P)	3 M, 6 M*, 12 M	T, IP
Psychological health and quality of life (secondary outcome measures)	PROMIS PROPr Short form (PROMIS-29 profile V2.1)	3 M, 6 M*, 12 M	T, IP, R
	Pittsburgh Sleep Quality Index (PSQI)	3 M, 6 M*, 12 M	T, IP, R
	NINDS Epilepsy Screening Questionnaire	3 M, 6 M*, 12 M	T, IP
	Rivermead Post-Concussion Questionnaire (RPQ)	3 M, 6 M*, 12 M	T, IP, R
	Patient health Questionnaire-9 (PHQ-9)	3 M, 6 M*, 12 M	T, IP, R
	Columbia Suicide Severity Rating Scale (C-SSRS) (only required if >1 on thePHQ-9 #9)	3 M, 6 M*, 12 M	T, IP
Abbreviated assessment battery for participants who are unable to take comprehensive assessment battery			
Consciousness and basic cognition	Confusion Assessment Protocol (CAP)	3 M, 6 M*, 12 M	IP
	Coma Recovery Scale Revised (CRS-R)	3 M, 6 M*, 12 M	IP
Global outcome	Functional Status Examination (FSE)	3 M, 6 M*, 12 M	T
	Revised Glasgow Outcome Scale Extended (R-GOSE)	3 M, 6 M*, 12 M	T, IP
	Expanded Disability Rating Scale Post-Acute Interview (E-DRS-P)	3 M, 6 M*, 12 M	T, IP

*Only for TBI group; T, telephone interview; IP, in-person interview; O, REDCap online survey.

GOAT, Galveston Orientation and Amnesia Test; NIH ToolBox- Cognitive Battery supplemented; HVLT, Hopkins Verbal Learning Test; WAIS-IV PSI, Processing Speed Index; SWAPS, Southwestern Assessment of Processing Speed; R- GOSE, Glasgow Outcome Scale Extended-Revised; FSE, Functional Status Examination; E-DRS-P Expanded Disability Rating Scale Post-Acute Interview; FIM, Functional Independence Measure; PROMIS PROPr, Patient-Reported Outcomes Measurement Information System; PSQI, Pittsburgh Sleep Quality Index; PTSD, Posttraumatic Stress Disorder; PCL-C, Checklist for Civilians; REDCap, Research Electronic Data Capture.

The participant will be counted as retained in the study when the outcome assessment battery is completed at 12 months post injury. Strategies will be used to maximize retention, such as: (i) during the consent, the research team will make sure that the participant and family fully understand what is involved in the study, (ii) the study coordinator will collect detailed contact information (i.e., primary and backup phone and email) from the family upon consent, and thorough completion of this measure will make future contacts more successful and maximize participant retention, (iii) scheduling follow-up visits at the most convenient time for the participant and using timely visit reminder strategies. Prior to the visit, we will send a confirmation letter including the appointment date, time, location and the duration of the appointment. We will use all available methods to contact the participant (e.g., phone, mail, e-mail, texting, contacting other people listed about how to reach the participant). Every effort will be made to complete the assessment during the visit window.

### Data collection

#### Clinical data

In order to standardize data collection and enable comparison, the collection of clinical variables will adhere to a predefined set of National Institutes of Health/National Institute of Neurological Disorders and Stroke Common Data Elements (NIH/NINDS CDEs). These variables encompass a wide range of information including demographics, social status, general health history, injury-related events, physical and neurological examination findings, laboratory test results, vital signs, clinical imaging findings on the initial head computed tomography (CT), hospitalization details, discharge information, and inpatient rehabilitation data. These variables will be collected through chart review and/or patient or family interviews (18, 19).

#### Data sharing plan

The data management and sharing plan will be consistent with the NIH Data Sharing Policy and Implementation Guidance. The Case report form and de-identified imaging data will be made available via the Federal Interagency TBI Research (FITBIR).

#### Physiological data

Cerebral hemodynamic data collection during the acute phase will commence within the first week (as soon as possible after the formal consent) of hospital admission for TBI patients, after which daily monitoring sessions will be performed throughout the first week post injury or until hospital discharge, whichever occurs first (Figure 1). For the OTC group, similar monitoring sessions will be performed as soon as possible after the formal consent.

All measures will be collected under resting conditions and the degree of head-of-bed elevation utilized as the standard of care for each individual will be documented since a patient’s head therapeutic positioning may have effects on cerebral hemodynamics (20).

**Figure 2:**
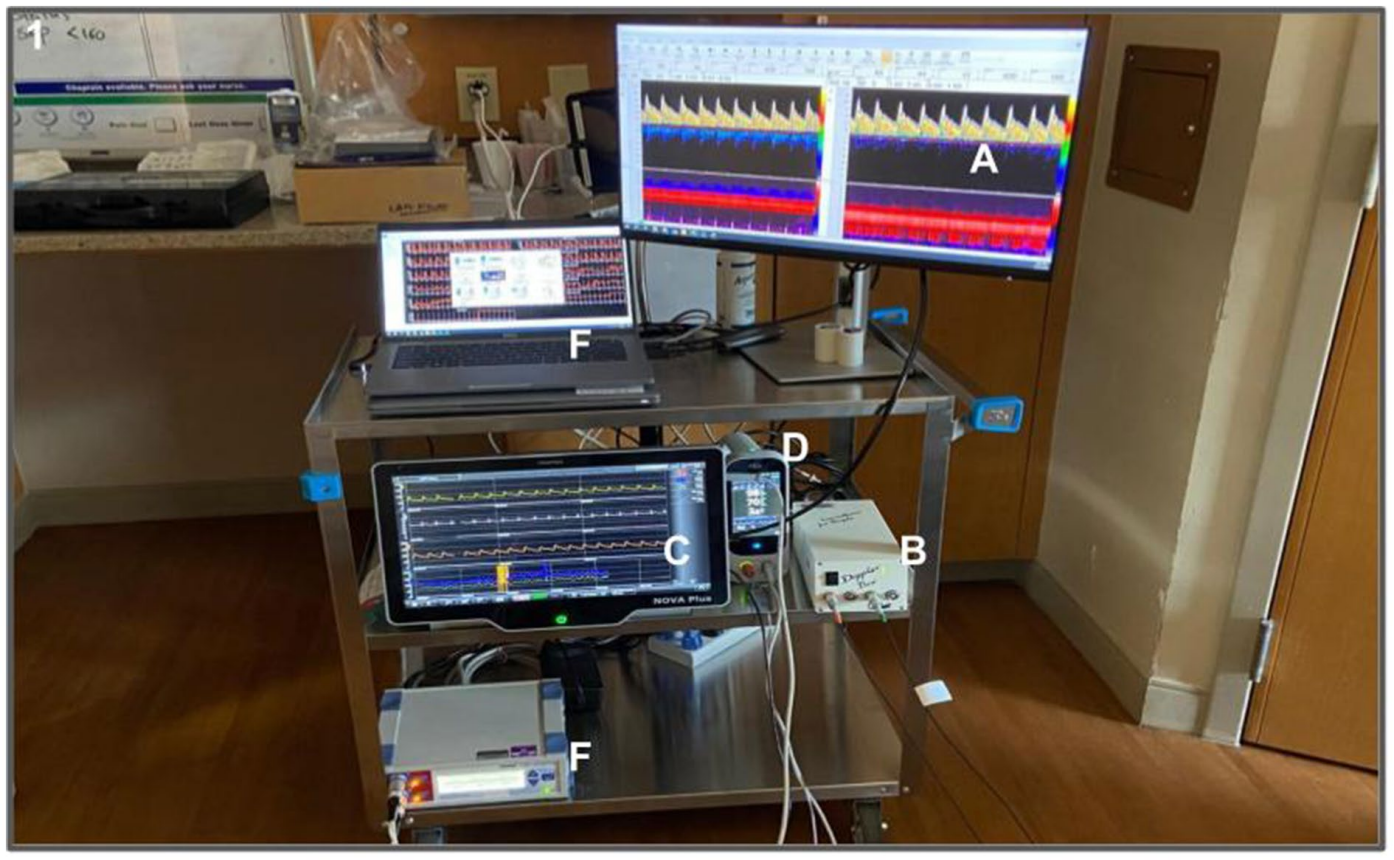
Intensive Care Unit bedside (ICU) physiological measurement set-up for CAPCOG study: ICU setting (A) cerebral blood velocity waveforms from Dopplerbox (V.10.5.1 software); (B) DWL Dopplerbox; (C) Finometer; (D) Saturimeter; (E) capnograph; (F) Near-Infrared Spectroscopy; (G) dedicated laptop for c data acquisition BIOPAC Systems.

Non-invasive arterial blood pressure (ABP) will be measured using a finger arterial photoplethysmography device (Finapres Nova, Finapres Medical Systems, The Netherlands), transcranial Doppler ultrasound (Multi-Dop T, DWL, Germany), near-infrared spectroscopy (moorVMS-NIRS, Moor Instruments, United kingdom), electrocardiogram (Finapres Nova, Finapres Medical Systems, The Netherlands), and capnography (Rad-97, Masimo, California, United States) to assess continuous beat-to-beat changes in ABP, cerebral blood flow velocity (CBFV), brain tissue oxygenation, R-R interval, breath-by-breath end-tidal CO_2_ (ETCO_2_) (see Supplementary Figure S1 and Figure 2). Additionally, if available, standard invasive intracranial pressure (ICP) and ABP measurements, as well as ETCO_2_ via mechanical ventilation and other clinically relevant signals, will be collected from the patient’s bedside monitor and integrated simultaneously with the non-invasive physiological signals using ICM+ software (Cambridge Enterprise Ltd., United Kingdom) (21). Monitoring sessions will last for 30 min, with all signals digitized and integrated via an analog-digital converter (DT9826; Data Translation, Marlboro, MA), and recorded using ICM+ software, with a sample rate of 500 Hz.

**Figure 3:**
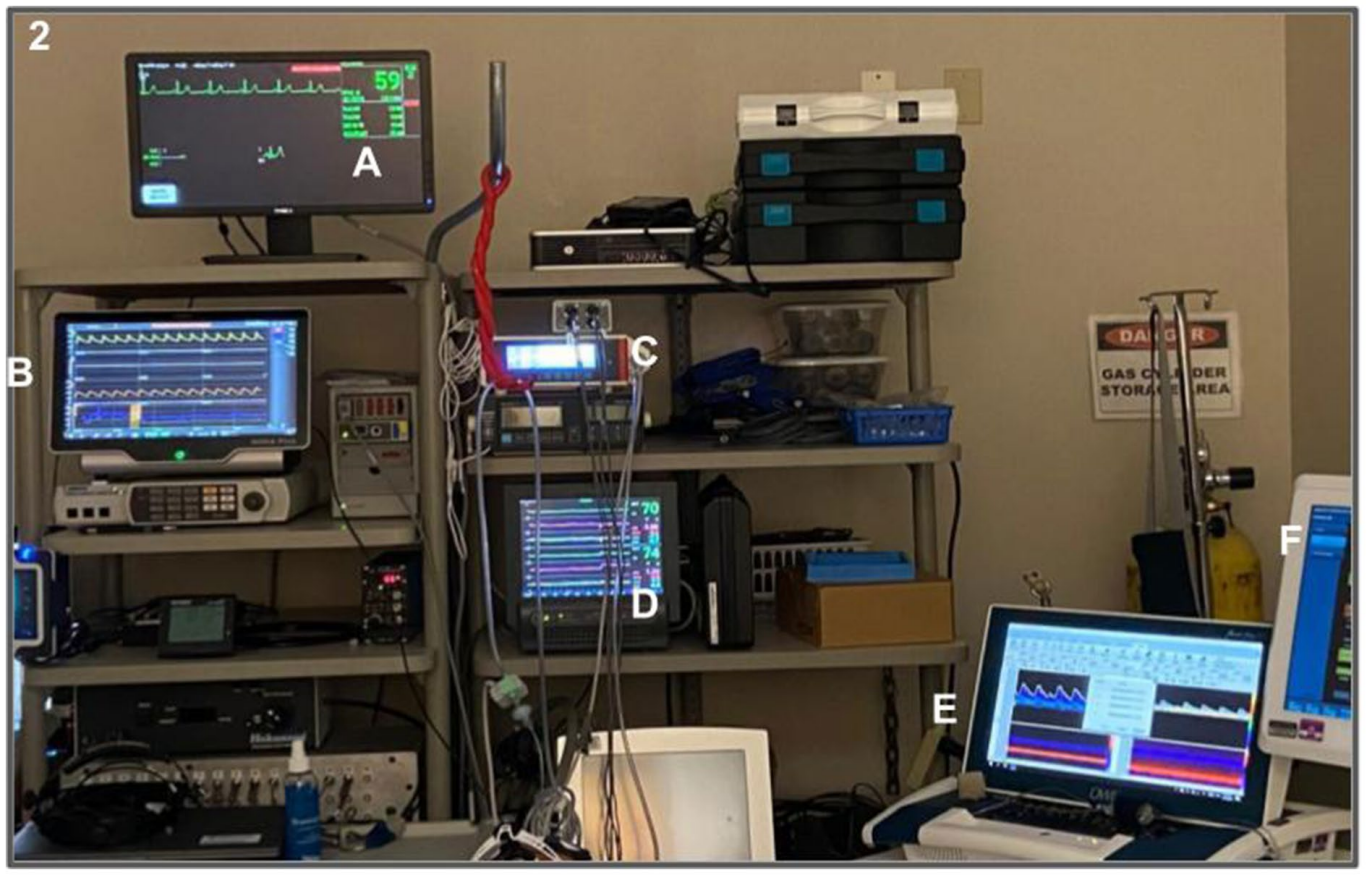
Follow up setting on the lab. (A) Electroencephalogram (B) Finometer; (C) capnograph; (D) Near-Infrared Spectroscopy; (E) Transcranial Doppler – DWL (F) Eletroencephalogram.

During follow-up, these above mentioned measurements will be repeated (Figure 3), with the addition of electroencephalogram (EEG) (Moberg CNS-300 monitor, Natus, Wisconsin, United States) and 2D color-coded duplex ultrasonography (CX-50, Philips, Massachusetts, United States) to measure volumetric cerebral blood flow (CBF) from the left and right internal carotid artery (ICA) and vertebral artery (VA). Total CBF will be calculated as the sum of blood flow measured from the bilateral ICA and VA. Additionally, cerebrovascular resistance (CVR) will be calculated as mean ABP divided by total CBF.

#### Physiological data processing

Cerebrovascular and cardiovascular data will be analyzed using AcqKnowledge (BIOPAC Systems, Goleta, CA), DADiSP (DSP Development Corporation, Newton, MA), MATLAB (MathWorks, Natick, MA) and ICM+ software (22, 23).

Dynamic CA assessed through the different monitoring modalities will be determined in two ways: time domain and frequency domain analyses. Time domain indices are based on the relationship between spontaneous changes in mean arterial pressure (MAP) and CBFV (10, 24–26).

Frequency domain analysis based on MAP-CBFV (TCD-derived), and MAP-Oxyhemoglobin (NIRS-derived) transfer functions will be computed bilaterally in each participant following an international Cerebrovascular Research Network (CARNet) guideline (22). Signals will be sampled (500 Hz) and stored for offline analysis. All signals will be filtered with an eighth-order Butterworth low-pass filter with a cut-off frequency of 20 Hz. The beginning and end of each cardiac cycle will be detected in the BP signal, and mean BP, CBFV and heart rate will be obtained for each heartbeat. Beat-to-beat parameters will be interpolated with a third-order polynomial and resampled at 5 Hz to generate signals with a uniform time base (22). Different TFA parameters (gain, phase, coherence) – will be calculated at very low frequency (VLF, 0.02–0.07 Hz), low frequency (LF, 0.07–0.2 Hz), and high frequency (HF, 0.2–0.5 Hz) ranges (27). Transfer function gain quantifies the magnitude relationship between input and output signals (such as systolic BP and MAP). The phase measures the temporal displacement between the input and output signals, and the coherence measures the strength of the linear relationship between the input and output signals (28).

A final averaged value from the bilateral TCD and NIRS measurements for each index will be calculated to assess *CA.* The CA of affected and unaffected hemisphere side by cranial CT scan will be also analyzed. We propose to use the transfer function phase as the primary measure of CA for its sensitivity and reliability to detect cerebrovascular dysfunction as demonstrated by our team and others (13).

Time domain analysis will be computed utilizing ICM+ software (10). A moving Pearson correlation coefficient calculated between systolic, diastolic, and mean CBFV and MAP using 30 consecutive 10-s windows will be used to derive the TCD-based CA indices systolic flow index (Sx_a) (10), diastolic flow index (Dx_a) (10), mean flow index (Mx_a) and autoregulation index (ARI) (29), respectively. NIRS-based CA indices will be calculated similarly, as the moving correlation between tissue oxygenation index and MAP (TOx_a) and that between tissue hemoglobin index and MAP (THx_a) (30, 31).

#### Imaging protocol

The initial clinical head CT data from the emergency room will be downloaded. Lesion characteristics will be documented in REDCap, including intracranial pathologies, subarachnoid hemorrhage, extra-axial or subdural hemorrhage, as well as findings related to cisterns and surgeries based on NIH Comment Data Elements. MRI scans will be collected at 3 months (±2 weeks) and 12 months (±1 month) post-injury, and analyses will account for the impact of lesion location and size on a subject-by-subject basis.

MRI data will be collected on a Siemens Prisma 3 T scanner: (1) 3D Multi-Echo T1-weighted Magnetization-Prepared-Rapid-Acquisition-of-Gradient-Echo (3D ME-MPRAGE) sequence (32) will be used to measure brain volumes. (2) 3D T2 FLAIR sequence (32) will be used to assess white matter hyperintensity volume and location (33). (3) 3D Multi-Echo Gradient Recalled Echo (3D ME-GRE) (32) will be used to visualize and quantify microbleeds and hemorrhage (34).

(4) 3D Pseudo Continuous Arterial Spin Labeling (3D PCASL) sequence will be used to measure regional CBF (35, 36). (5) Diffusion tensor imaging (DTI) with multiple b values, including 114 diffusion-weighted image volumes, will be used to assess white matter microstructural integrity (37). (6) Resting-state fMRI (rs-fMRI) will be used to assess brain network functional connectivity with a focus on the DMN (Default Mode Network) (38–42).

Each MRI scan will be visually reviewed within three business days. Preliminary processing will generate maps including FreeSurfer T1 segmentation, R2*, SWI, QSM, ASL perfusion, DTI FA/MD, fiber tracking, and resting-state fMRI SNR/connectivity. Dr. Zhu (MRI physicist) or his trainee will assess image quality. Inferior image quality is often due to motion, acquisition errors, or system issues. Imaging sequences with inferior quality will be marked, and rescanning will be attempted if feasible. Poor-quality sequences will not be included in final analyses.

##### TBI lesion characterization

A neuroradiologist will visually review T1, T2 FLAIR, and GRE images (the R2*, SWI and QSM maps genrated from the multi-echo GRE images as well) to assess “lesion load.” Participants with large lesions (>5 cm^3^ subcortical, >50 cm^3^ cortical) will be excluded from automated analysis, but DTI metrics and CBF will be examined on these lesions. Case-by-case approaches will address large lesions.

##### Volumetric data processing

3D T1 MPRAGE images will undergo a series of FreeSurfer-based processing procedures to generate cortical and subcortical morphometrics (43, 44). To improve the cortical surface reconstruction, T2 FLAIR data will be incorporated into FreeSurfer’s reconstruction pipeline (45). To reduce the potential impact of brain lesions due to TBI on cortical surface reconstruction, we will visually inspect each segmentation and manually label and remove vertices on the cortical surfaces caused by the lesions (45). The scans which fail to complete the processing pipeline or require major manual edits will be excluded for further analysis (45). The corrected maps and regional volumes will be used for further post-processing.

#### rs-fMRI data processing

We include rs-fMRI as an exploratory measure of changes in brain neural network connectivity after TBI (40). AFNI (Analysis of Functional NeuroImages) will be used for rs-fMRI data preprocessing (55). A robust independent component analysis based preprocessing strategy will also be assessed (40). The DMN functional connectivity has been demonstrated to be an important marker in AD (41) and TBI (56). The nodes of the DMN network as well as other networks can be extracted with the atlas published by Yeo et al. (57) in young adults or by Shirer et al. in older adults (58). The overall DMN functional connectivity, and similarly for other networks, at each time point will be calculated as the average of the pair-wise Pearson correlation coefficients of all connection pairs of DMN nodes and will be used for within-subject as well as for group comparisons (40).

##### Image analysis integration

All MRI images will be aligned to the T1 MPRAGE images which will be standardized to the MNI305 space via the FreeSurfer processing pipeline (46). Primary analysis will be carried out in the subject space. Voxel-based group analysis will be carried out in the MNI space.

##### Arterial spin labeling (ASL) data processing

CBF maps will be generated from pCASL sequence based on the equation proposed by the ISMRM Perfusion Study Group (35, 47).

##### DTI data processing

The DTI data will be processed with FSL (48). The “topup” procedure will be used to correct susceptibility distortions (49). The Track-Based Spatial Statistics program (50) will be used to estimate fractional anisotropy, mean diffusivity, axial diffusivity, and radial diffusivity (51, 52). Free water content will be estimated with the DIPY software package (53). Probabilistic tractography will be performed to estimate brain network structural connectivity (40, 41, 54).

#### Functional and cognitive outcomes at 3, 6, and 12 months

A detailed neuropsychological assessment will be administered by research staff trained and supervised by a neuropsychologist at all three followup time points and will take approximately 2.5 to 3 h. The screening assessment, including the GOAT and speech intelligibility, will be evaluated either by phone or in person (Table 2).

#### Cognitive outcomes

This assessment will include primary outcome measures that tap into a variety of cognitive domains in person during the follow up visit, as reflected by various measures in the computerized National Institutes of Health (NIH) Toolbox. The NIH Toolbox Cognitive Battery, administered via iPad, includes 7 subtests to assess executive function, attention, episodic memory, working memory, processing speed, and language. We also will perform the Hopkins Verbal Learning Test (HVLT) to assess episodic memory, the Trail Making Test for attention, speed, and mental flexibility and Processing Speed Index (WAIS-IV PSI) and SWAPS (aka TAPS).

#### Functional outcomes

Secondary outcome measures will include the Glasgow Outcome Scale Extended-Revised (R-GOSE) (59), Functional Status Examination (FSE), Expanded Disability Rating Scale Post-Acute Interview (E-DRS-P), and Functional Independence Measure (FIM).

#### Psychological health and quality of life

Psychological health, sleep, stress, and quality of life will also be evaluated using various measures popular measures including the PROMIS PROPr Short form (PROMIS-29 Profile V2.1), Pittsburgh Sleep Quality Index (PSQI), and PTSD Checklist for Civilians (PCL-C), among others (Table 2).

Furthermore, for participants who are unable to take Comprehensive Assessment Battery the abbreviated Assessment Battery will be assessed within 1–15.5 h using the Confusion Assessment Protocol (CAP), Coma Recovery Scale-Revised (CRS-R), R-GOSE, FSE, Expanded Disability Rating Scale Post-Acute Interview (E-DRS-P), and FIM (Table 2).

#### Data management and quality assurance

A REDCap database will be developed to collect and store data (60). The REDCap database will be hosted on a secure, HIPAA compliant server at the University of Texas Southwestern Medical Center. The study coordinator will use REDCap’s data quality control module to report missing values, validation errors, and outliers, as well as run simple reports using REDCap. All MRI subject data will be collected without identifiable subject information and will be uploaded to a secured data server.

### Power and sample size

This study is powered to examine the relationship between the primary outcome of NIHTB_CB fluid composite score at 1-year postinjury and dynamic CA indices derived from frequency-domain analysis during the acute stage of TBI (i.e., <1-week postinjury). According to our previous study (13), the dynamic CA transfer function phase at low frequency range was positively correlated with NIHTB_CB fluid composite score (i.e., a higher phase, a better CA, was associated with better cognitive score), with the coefficient of determination ( $R^{2}$ ) of 0.35 (or Pearson correlation coefficient = −0.593). Based on this information, it is estimated that 117 participants (i.e., a cohort of TBI *n* = 90 and orthopedic controls *n* = 27) will provide 82% power at a 5% two-sided significance level to detect a 0.053 increase in $R^{2}$ attributed 2 additional dynamic CA variables (transfer function gain and coherence), adjusting for 6 confounding factors (initial GCS, posttraumatic amnesia, head CT scan, age, sex, and education) in a multiple regression model, where $R^{2}=$ 0.35 when only the control factors are included in the model (NCSS PASS 16). With a 10% attrition rate, the total sample size of *n* = 130 participants (TBI = 100, control = 30) are required to be enrolled for this study.

### Statistical analysis plan

Discrete variables will be summarized using counts with percentages, and continuous variables using means with standard deviation or medians with interquartile ranges. The comparisons between TBI and OTC groups in dynamic CA indices during the acute phase will be evaluated using a two-sample t-test or nonparametric Wilcoxon test. No adjustment will be made for multiple comparisons, and therefore findings for secondary outcomes should be interpreted as exploratory. Two-sided 0.05 level of significance will be used for statistical hypothesis testing.

#### Statistical methods: outcomes

For the primary analysis, multiple linear regression models will be performed to examine the associations of dynamic CA indices on the primary outcome of NIHTB_CB fluid composite score at 1 year post injury, adjusted by potential confounders (i.e., initial GCS, posttraumatic amnesia, head CT scan, age, sex, etc.). The parsimonious model will be selected via the automatic model selection algorithms, including forward, backward, and stepwise selection. Multicollinearity will be evaluated using variance inflation factors, and the issue of overfitting will be assessed through 10-fold cross-validation. For the secondary analyses, secondary outcomes, including CA/CBF and other continuous cognitive measures, R-GOSE score as well as health-related PROMIS measures at 1-year postinjury, will be analyzed separately through the same analytical approach. Sensitivity analyses of the outcomes will be performed to assess the robustness of the findings, including model diagnostics, outlier assessments, distributional assumptions, and missing data. Data transformation will be made if the normality assumption is violated.

#### Statistical analysis: additional analyses

Mixed-effects models with an unstructured covariance matrix will be used to estimate the primary outcome of brain volumes measured at 3 and 12 months postinjury; the models will include subjects as random effect and dynamic CA indices, time, and dynamic CA indices × time interaction as fixed-effects factors. Other covariates or potential confounders (e.g., age, sex, dynamic CA indices and others) will be included in these mixed-effects models. Conditional Akaike information criteria and other goodness of fit criteria will be applied to select the parsimonious mixed-effects model. Similar modeling and data analysis will be applied with DTI measure of white matter integrity as a dependent variable. In the secondary analysis, mediation models will be performed to study the overall association of Δ CA and CBF variables (both global CBF measured by 2D color-coded duplex ultrasonography and regional CBF measured by ASL) with the outcome of Δ NIHTB_CB fluid composite score and to estimate the extent to which this association is mediated by Δ brain volumes or white matter integrity. We will repeat the above procedures for the DMN connectivity from rs-fMRI to explore if the associations between cerebrovascular function recovery, cognitive and functional outcomes are mediated by changes in brain neural network connectivity. Secondary outcomes, including changes in cognitive outcomes, R-GOSE score and PROMIS measures, will be analyzed followed by similar analytical approaches, and evaluated both total effects of Δ CA/CBF variables and mediation effects of Δ brain volumes. No adjustments for multiple comparisons will be made for all secondary analyses, which are considered as exploratory analyses. Assumptions and diagnosis for mixed-effects models will be carefully evaluated. Missing data will be imputed using multiple imputation by chained equations and pattern mixture approaches as needed and evaluated by the sensitivity analyses.

### Potential risks

The proposed project is an observational study. Every effort will be made by the study investigators to minimize possible risks of psychological and/or physiological discomfort of subjects during either interviews or studies by reassurance of the subjects and by offering rest breaks if needed or required. The members of the research team are experienced in all procedures proposed in this study.

There are no known risks to using the techniques (i.e., TCD, NIRS and others) in human subjects. All neuropsychological evaluations will be held in confidence and shared with the patient and family at the end of the evaluation and when diagnostic category is changed during the follow-up examination. In addition, all subjects will be directly observed during the imaging procedure and are in constant communication with the MRI technician. Should a participant experience discomfort during a procedure, the study will be stopped.

#### Adverse event (AE) and serious adverse event (SAE) reporting

An Adverse Event is any adverse change from the participant’s baseline condition, including clinical or laboratory abnormalities occurring after study consent. Serious Adverse Events include events that are fatal, life-threatening, significantly disabling, result in hospitalization, prolong hospital stays, or are associated with congenital abnormalities or birth defects. Additionally, any experience considered serious by the investigator, or indicating significant hazard, contraindication, side effect, or precaution related to study participation, will be reported as an SAE. The participants will be overseen by physicians and research staff responsible for assessing and reporting AEs and SAEs according to established NIH and University of Texas Southwestern Institutional Review Board (IRB) guidelines. All subjects reporting AE and SAE will be evaluated and treated by physicians as needed. All AE and SAE will be reported to the IRB, NIH, and FDA (when required) to ensure participant safety.

## Discussion

CAPCOG_TBI study is an observational study that will investigate long- term cognitive outcomes after msTBI at three level 1 trauma centers in Texas, USA. The main purpose of the study is to determine if acute cerebrovascular dysfunction after msTBI is associated with cognitive outcome at 1-year post-injury. To our knowledge, no prospective studies have evaluated cerebral vascular assessment in the acute phase and compared it with long-term cognitive outcomes in msTBI patients.

The rationale for the CAPCOG study arises from the significant variability in clinical outcomes and brain lesions resulting from trauma, which can range from no measurable impairment to a vegetative state (12, 61). This variability presents a considerable challenge in predicting and prognosticating cognitive outcomes following msTBI.

Previous studies have explored relationships between cerebrovascular dysfunction and cognitive outcomes at 6 and 12 months post-TBI (10, 62). However, these analyses were performed retrospectively. Unfortunately, the largest prospective studies published on this topic, analyzing long-term function outcomes, did not include cerebrovascular assessments (3, 11). Nonetheless, the acute cerebrovascular dysfunction assessed by CA in TBI may play a role in cognitive outcomes and prediction models. Therefore, this large longitudinal prospective study meets the urgent need to understand temporal associations of CA recovery with functional and cognitive recovery during the critical time window of 12 months after TBI.

Another point to raise is that the majority of studies assessing functional outcomes have relied exclusively on the GOSE, which is a crude measure of outcome. Robust use and the combination of more detailed cognitive assessments and functional scales are crucial for a comprehensive analysis of cognitive function outcomes, such as NIHTB-CB, RAVLT, WAIS-IV PSI, FIM, and PROMIS PROPr scales. To mitigate potential limitations in cognitive assessment, all of these scales will be employed in the CAPCOG_TBI study.

In agreement with initial retrospective studies (10, 62), we expect that the severity of cerebrovascular dysfunction during the acute stage will be inversely associated with cognitive and functional outcomes in msTBI. Furthermore, another important point to address is the temporal recovery of cerebral vascular function and its relationship with cognitive outcomes. A recent study REF investigated the daily course of CA over the first 10 days post-TBI, revealing a significant temporal association between impaired CA, particularly days 2–5 post-injury, and poor outcomes at 6 months was reported (63). The CAPCOG_TBI study aims to further assess the temporal association between the recovery of CA and cognitive function over the long term, at 3, 6, and 12 months of follow-up.

Temporal associations of acute cerebrovascular dysfunction and its recovery also will be evaluated with imaging biomarkers of neurodegeneration after TBI. Changes in cerebrovascular function and brain structure may correlate with cognitive and functional outcomes. Including brain structural changes in the mediation path may mitigate the associations between cerebrovascular function and cognitive and functional outcomes. MRI and cerebral vascular assessments will be performed during follow-up evaluations to elucidate the temporal trajectory and its association with cognitive outcomes. The rich longitudinal physiological and imaging data collection will provide insight in the potential mediating role of cerebrovascular dysfunction in the post-TBI neurodegeneration process.

Some of the inherent limitations of the study lie in its design. To reduce selection bias, patient inclusions must be consecutive, which will be addressed with daily screening for potential participants. Another potential issue is the loss of follow-up, which can introduce bias and compromise the validity of the results. To address this, we will implement a comprehensive follow-up retention plan. Strategies to maximize retention will include appointing a dedicated study coordinator for this project. The recruitment and screening will be conducted daily by a team with extensive experience in recruiting TBI patients during both the acute and chronic stages for research studies at two trauma centers. Additionally, in long-term prospective studies, there is a risk of changes in technologies and diagnostic methods over time. To reduce this possibility, we are employing state-of-the-art procedures and methodology that has been exhaustively studied and validated in previous publications. One notable limitation of our analyses is that we do not plan to apply adjustments for multiple comparisons with respect to secondary outcomes and analyses. This approach is intentional due to the exploratory nature of the study, which aims to identify potential associations and generate hypotheses for future research. Consequently, this means that our findings should be interpreted with caution, as the risk of Type I error is increased. We have highlighted this limitation to underscore the need for further research to confirm and validate these preliminary results.

## Conclusion

In conclusion, due to the complexity of TBI lesions/dysfunction and their consequences, and the current lack of literature regarding acute cerebral dysfunction with long-term cognitive outcomes, the CAPCOG_TBI study was developed. A better understanding of the heterogeneity of traumatic brain lesions through cerebrovascular assessments could pave the way for innovative vascular-focused interventions aimed at enhancing cognitive recovery and mitigating neurodegeneration following msTBI.

The Institutional Review Board approved this study, STU-2023-0254, and ensured that written informed consent to participate will be obtained from all participants.

## Supplementary material

FIGURE S1 The figure provides a detailed illustration of the participant timeline. msTBI, moderate-severe trauma brain injury; TCD, transcranial doppler; ABP, arterial blood pressure; Near infrared spectroscopy; US, ultrasound; EKD, electrocardiogram; EEG, electroencephalogram; MRI, Magnetic Resonance Imaging. * MRI will be performed at 3 and 12 months. **6 months follow-up will be only done in TBI group.
