# Supplementary figures and images for: Study protocol: Cerebral autoregulation, brain perfusion, and neurocognitive outcomes after traumatic brain injury -CAPCOG-TBI
Source: Front Neurol. 2024 Oct 16;15:1465226. doi: 10.3389/fneur.2024.1465226 (PMC11521900; doi:10.3389/fneur.2024.1465226)

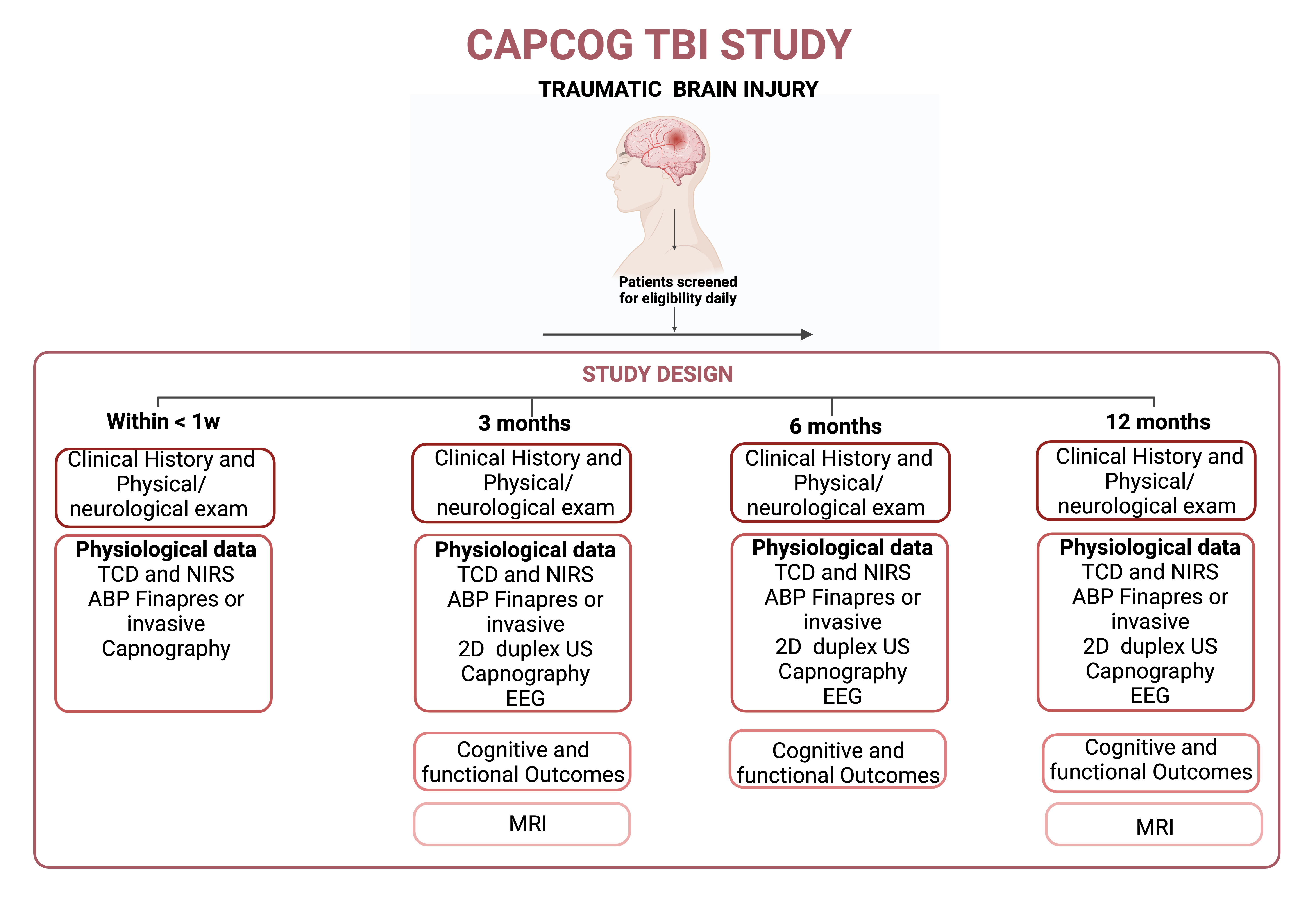

Supplement: FIGURE S1 — The figure provides a detailed illustration of the participant timeline. msTBI, moderate-severe trauma brain injury; TCD, transcranial doppler; ABP, arterial blood pressure; Near infrared spectroscopy; US, ultrasound; EKD, electrocardiogram; EEG, electroencephalogram; MRI, Magnetic Resonance Imaging. * MRI will be performed at 3 and 12 months. **6 months follow-up will be only done in TBI group. [file Image_1.JPEG]
